# C-Reactive Protein and Arteriosclerosis

**DOI:** 10.1155/2014/646817

**Published:** 2014-05-26

**Authors:** Jan Torzewski, Jianglin Fan, Heribert Schunkert, Alexander Szalai, Michael Torzewski

**Affiliations:** ^1^Cardiovascular Center Oberallgäu-Kempten, Robert-Weixler Straße 50, 87439 Kempten/Allgäu, Im Stillen 3, 87509 Immenstadt, Germany; ^2^Department of Molecular Pathology, School of Medicine, University of Yamanashi, Chuo-City, Yamanashi 409-3898, Japan; ^3^Deutsches Herzzentrum München and Technische Universität München, Deutsches Zentrum für Herz-Kreislauf-Forschung, Munich Heart Alliance, Munich, Germany; ^4^Division of Clinical Immunology and Rheumatology, Department of Medicine, The University of Alabama at Birmingham, Birmingham, Al 35294-2182, USA; ^5^Department of Laboratory Medicine, Robert Bosch Hospital, Auerbach Street 110, 70376 Stuttgart, Germany

Although extensively studied and indeed emotionally discussed for more than two decades the role of C-reactive protein (CRP) in cardiovascular disease remains controversial. Three major questions are still not yet resolved. (1) Is CRP a clinically relevant marker of cardiovascular risk? (2) Is CRP even more than a risk marker, that is, a risk factor in cardiovascular disease? (3) Finally, is CRP a cardiovascular drug target?

This special issue comprises four review articles and three research articles that reflect the ongoing controversy over the subject. The review articles discuss CRP as a cardiovascular risk marker, CRP in animal models, CRP in its native and nonnative conformation, and CRP in human arteriosclerosis. The research articles deal with CRP as a cardiovascular risk marker in non-ST elevation acute coronary syndrome and with CRP as a drug target. The discussion climaxes in two research articles describing the use of CRP specific antisense oligonucleotides (ASOs) for the treatment of cardiovascular disease in animal models. The conclusions are indeed contradictory. When looking at the two articles, three general points may have to be taken into consideration. (1) Is the statistical power of the animal experiments adequate to draw definitive conclusions? (2) Are there off-target effects of the ASOs that may confound the results? And (3) is it necessary to design studies incorporating multiple on-target ASOs?

Specific CRP inhibition followed by use of CRP inhibitors in controlled clinical trials may be the only way to prove or disprove a causative role for CRP in cardiovascular disease.

